# Improving peripheral arterial catheter placement in critically ill children: a pre-post quality improvement study

**DOI:** 10.3389/fped.2026.1931589

**Published:** 2026-08-25

**Authors:** Min Zhou, Kunhui Fu, Jiajia Li, Qiaoxia Wang, Na Yang, Liting Zeng, Linjuan Wang

**Affiliations:** Pediatric Intensive Care Unit, Shenzhen Children's Hospital, Shenzhen, China

**Keywords:** peripheral arterial catheter, pediatric intensive care unit, quality improvement, nursing adherence, pediatric nursing, ultrasound guidance, statistical process control

## Abstract

**Introduction and aims:**

Peripheral arterial catheter (PAC) assessment and insertion are essential but technically demanding procedures in pediatric intensive care. Practice varies widely, and inconsistent adherence to evidence-based standards contributes to preventable complications. This study aimed to improve the quality and safety of arterial catheterization in critically ill children through a structured quality improvement approach.

**Methods:**

A pre-post quality improvement study was conducted in a pediatric intensive care unit (PICU) across three phases: baseline (April–September 2024), implementation (October 2024–February 2025), and follow-up (March–September 2025). Phase-specific samples comprised 121, 76, and 63 patients and 170, 105, and 100 catheterizations, respectively; patients could contribute to more than one phase. A structured audit-and-feedback system measured adherence to 12 operational audit criteria derived from 10 retained evidence-based recommendations. Barriers to best practice were identified through stakeholder interviews, and a multi-component intervention bundle—incorporating standardized checklists, ultrasound-guided cannulation training, electronic decision support, and tiered competency assessment—was developed and implemented. Statistical process control charts were the primary longitudinal analysis, with phase comparisons as supportive analyses.

**Results:**

Overall nursing adherence increased from a baseline centerline of 57.0% to a post-implementation centerline of 74.3% (baseline-to-implementation *p* < 0.001). Assessment adherence increased from 69.4% to 88.9%, and insertion adherence increased from 36.6% to 53.6% (*p* < 0.01). First-attempt success changed only from 64.1% to 68.3%, without a sustained statistical process control shift or significant phase differences (all *p* > 0.20). Complication rates showed a significant downward trend (Kendall’s Tau = −0.432, *p* = 0.017).

**Conclusions:**

The quality improvement bundle was associated with improved process adherence and lower observed complication rates in pediatric PAC management. However, the uncontrolled design limits causal attribution for the safety outcome. Assessment adherence responded rapidly, whereas insertion performance and first-attempt success remained operator-dependent, supporting continued competency-based training and routine ultrasound guidance.

## Introduction

Peripheral arterial catheterization (PAC) is a common procedure in the pediatric intensive care unit (PICU), used for continuous hemodynamic monitoring, real-time arterial blood gas analysis, and repeated blood sampling in critically ill patients (1). Compared with intermittent non-invasive blood pressure monitoring and repeated peripheral venipuncture, PAC provides greater accuracy, reduces puncture frequency, and minimizes pain and discomfort (2).

PAC is an invasive procedure, however, and carries a real risk of complications—catheter occlusion, puncture site bleeding, and local hematoma—that can affect patient safety (3). A retrospective cohort study of 228 critically ill children found an overall complication rate of 33%, with catheter dysfunction (59%), bleeding (16%), leakage (8%), and hematoma (4%) as the most common events (4). Multiple puncture attempts and the use of multiple insertion sites were independent predictors of catheter dysfunction (4). Beyond operator factors, variability in staff experience, differences in knowledge levels, and the absence of standardized protocols for PAC insertion and maintenance all contribute to inconsistent practice across PICUs (5, 6). International guidelines (7) and consensus recommendations (8) exist, but their adoption into routine practice remains uneven (9).

Audit and feedback is one of the most widely studied strategies for changing clinical behavior. Systematic reviews have shown that structured audit cycles—measuring current practice against evidence-based criteria, then feeding results back to clinicians—can produce meaningful improvements in adherence (10, 11). This approach has been applied successfully across a range of clinical settings to standardize care processes and improve patient outcomes (12). In arterial catheterization management in pediatric critical care, however, such approaches remain rare.

The motivation for this study came from both global and local concerns. Children in the PICU are particularly vulnerable to catheter-related complications because of their anatomical and physiological characteristics (13). Procedural complexity, frequent staff turnover, and variable training all contribute to inconsistent practice. In our institution—a tertiary pediatric hospital in southern China—internal quality monitoring showed suboptimal adherence to existing arterial catheterization protocols and complication rates exceeding national benchmarks. Frontline nurses reported a lack of structured decision-support tools, and variations were observed in catheter size selection, insertion site assessment, infection control practices, and documentation. These observations are consistent with previous studies calling for structured interventions tailored to pediatric critical care (14).

This study was conducted in the PICU of a tertiary pediatric hospital in Shenzhen, China. The aim was to implement a structured quality improvement approach to improve the quality and safety of arterial catheterization in critically ill children—identifying best-practice recommendations for PAC assessment and insertion, implementing targeted strategies, and evaluating their impact on clinical practice and patient outcomes.

## Objectives

The primary objective was to implement a multi-component quality improvement bundle for PAC assessment and insertion in critically ill children and to evaluate its impact on nursing adherence, first-attempt success, and complication rates. Specifically, the study aimed to:

1. Determine baseline compliance with evidence-based recommendations for PAC assessment and insertion in the PICU.
2. Identify barriers and facilitators affecting compliance, and develop targeted strategies to address areas of non-compliance.
3. Implement these strategies and evaluate their impact on compliance and PAC-related complication rates.
4. Improve nurses’ adherence to standardized procedures and sustain improvements in PAC practice.

## Methods

### Study design and setting

**Figure 1:**
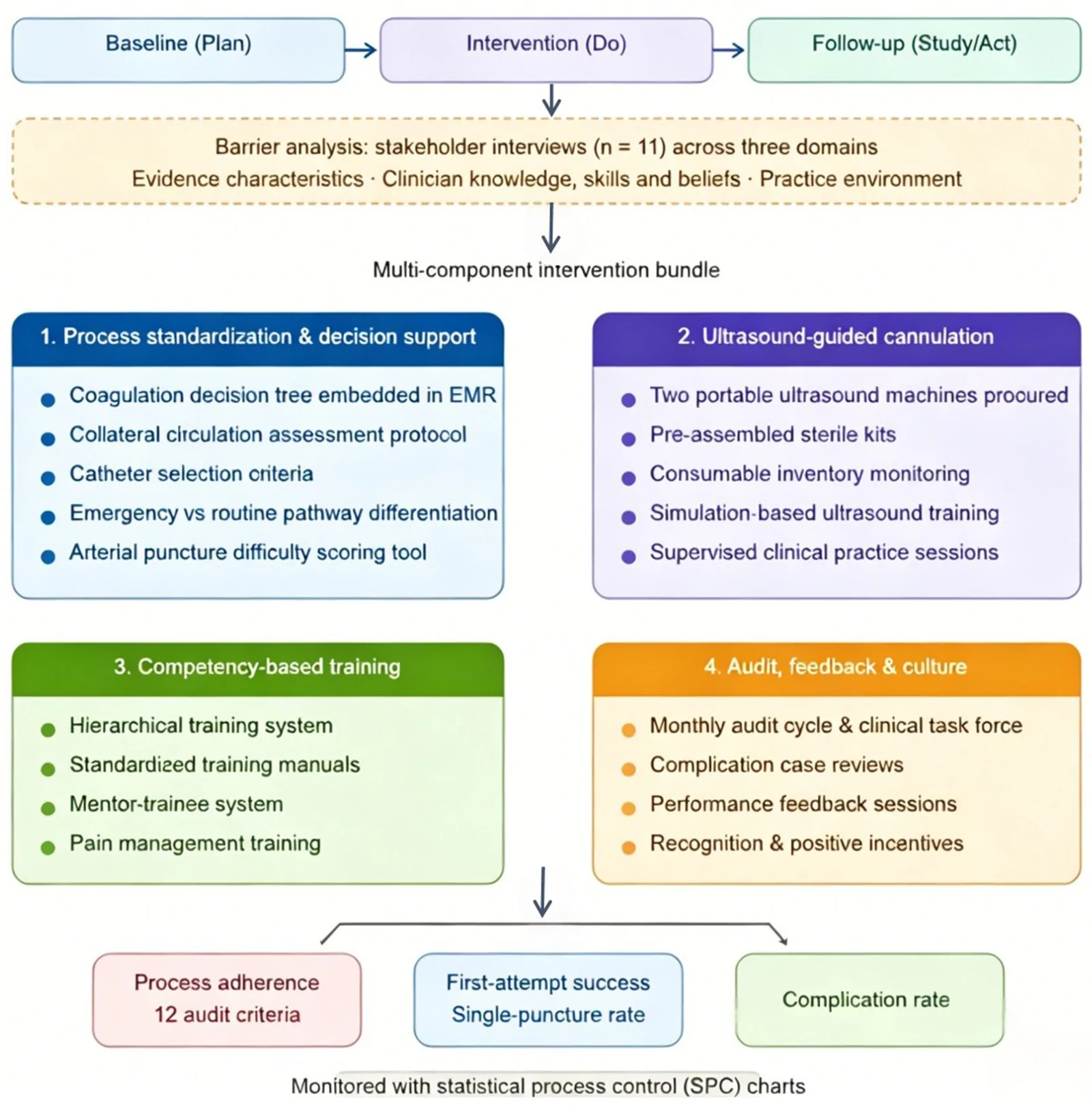
Multi-component quality improvement intervention framework for pediatric PAC management.

This pre-post quality improvement study was conducted in the 30-bed PICU of a tertiary Grade A pediatric hospital in Shenzhen, China, from April 2024 to September 2025. The unit is staffed by a multidisciplinary team of intensivists, anesthesiologists, 40 specialist nurses, and infection control physicians. The study comprised three consecutive phases: baseline (April–September 2024), implementation (October 2024–February 2025), and follow-up (March–September 2025). The study design is shown in Figure 1.

### Project team

A seven-member project team was assembled: one nursing department director (institutional oversight), one PICU head nurse (quality assurance and resource coordination), two specialist nurses (education and data collection), two unit team leaders (on-site audit and communication), and one research nurse (data analysis and manuscript development). Interdisciplinary meetings with PICU physicians, hospital quality managers, and frontline nursing staff were held to identify contextual barriers, validate audit criteria, and co-develop feasible strategies.

### Baseline audit

A previously published evidence summary on peripheral arterial catheter placement and management in critically ill children (15) served as the basis for developing the audit criteria. Eleven evidence-based recommendations covering indications, contraindications, coagulation status evaluation, site selection, catheter sizing, insertion technique, infection prevention, and pain management were extracted. After expert assessment of feasibility, appropriateness, and clinical relevance, 10 recommendations were retained and operationalized into 12 clinical audit criteria (Supplementary Appendix Table 1). The strength of each recommendation was graded using a standardized evidence classification system (16).

A baseline audit was conducted from April to September 2024. All auditors completed standardized training on the audit criteria, observation procedures, and judgment definitions. A calibration session was held before data collection to align inter-rater interpretations. Two trained auditors who were not involved in catheter insertion independently observed and recorded compliance with the twelve audit criteria during PAC procedures. First-attempt success rates and procedure-related complications were also recorded. Arterial catheter insertions were performed by qualified PICU nurses.

### Identifying barriers to best practice

To understand why adherence to best-practice recommendations was suboptimal, semi-structured interviews were conducted with 11 key stakeholders—nurse managers, unit team leaders, specialist nurses, senior registered nurses, and staff nurses. Eligibility required at least three years of PICU clinical experience and independent performance of a minimum of five arterial catheterizations per month. The interview guide explored three areas: (1) the evidence and recommendations themselves (complexity, clarity); (2) clinician-related factors (knowledge, skills, beliefs, attitudes); and (3) practice environment factors (workflow, resources, organizational support, culture).

Several enabling conditions were also identified. A strong team culture and high intrinsic motivation among nurses—particularly toward ultrasound-guided cannulation—provided a favorable climate for change. Leadership support from the head nurse and unit management reinforced institutional priority for vascular access improvement. Additional facilitators included improved access to ultrasound equipment, informal mentoring from experienced clinicians, and planned enhancements to the electronic medical record (EMR) system for decision-support integration.

### Multi-component intervention

Based on the barriers and facilitators identified, a multi-component quality improvement intervention was developed (Supplementary Appendix Table 2; Figure 1). The intervention bundle comprised four domains.

#### Process standardization and decision support

A coagulation assessment decision tree was developed and embedded into the EMR with automated reminders. Standardized protocols mandated assessment of collateral circulation and coagulation status before PAC insertion and established clear criteria for catheter selection. An arterial puncture difficulty scoring tool and simplified operating procedures were introduced. Emergency and routine care pathways were differentiated to balance timeliness with safety.

#### Ultrasound-guided cannulation

Two portable ultrasound machines were procured, and a consumable inventory monitoring mechanism was established. Pre-assembled sterile ultrasound kits and pre-positioned machines reduced setup time and minimized workflow disruption. Simulation-based training with supervised clinical practice built competence in ultrasound-guided techniques.

#### Competency-based training

A hierarchical training system incorporated simulation-based practice, real-scenario assessments, and supervised clinical procedures. Only nurses with more than three years of PICU experience were authorized to independently perform ultrasound-guided catheterization. Standardized training manuals and a mentor-trainee system supported ongoing skill development. Training on pain management emphasized topical anesthesia, patient comfort positioning, and minimizing restraint use.

#### Audit, feedback, and culture change

A clinical task force conducted monthly audits and proposed iterative improvements. Monthly complication case reviews and performance feedback sessions were implemented. Recognition mechanisms and positive incentives reinforced adherence to best practices. A data feedback loop enabled continuous monitoring and timely corrective action.

### Follow-up audit and sustainability

During the implementation phase (October 2024–February 2025), the multi-component intervention was implemented, and an implementation-phase audit was conducted using the same criteria and procedures as in the baseline phase, allowing direct comparison of adherence, first-attempt success, and complication rates.

A sustainability phase (March–September 2025) evaluated whether improvements were maintained. Key audit indicators were incorporated into unit protocols and the EMR system. Monthly audits continued, with findings discussed at team meetings. The tiered training system—including simulation, mentorship, and refresher sessions—was institutionalized to ensure sustained competence among new staff. Performance recognition and case sharing continued through monthly quality meetings, supported by a vascular access task force.

### Outcome measures

Three outcome domains were evaluated: process adherence, first-attempt success, and complication incidence.

Process adherence was assessed through compliance with the twelve PAC audit criteria, using direct observation and documentation review; each criterion was recorded as compliant or non-compliant. First-attempt success was defined as successful catheter insertion on the initial puncture without reattempts. Complication incidence included local bleeding, hematoma, and leakage within 24 h post-insertion.

#### Data collection

Data were collected across all three phases using standardized procedures. Gender was recorded as a patient-level characteristic. Age, body mass index (BMI), body weight, primary diagnosis, Pediatric Critical Illness Score (PCIS) (17), catheter site, laboratory parameters obtained within 24 h before catheter insertion, and mean arterial pressure (MAP) at arterial puncture were recorded for each catheterization. The PCIS corresponding to the relevant PICU admission was obtained from the assessment completed within the first 24 h of that admission. Records were retrieved through the nursing information system and institutional laboratory platform. Data collection procedures remained consistent across phases.

An electronic database was established with real-time data entry and cross-checking. Data were independently entered and verified by project team members. The study protocol was approved by the institutional ethics committee, and all data were anonymized before analysis.

## Statistical analysis

All statistical analyses were performed using R software (version 4.5.1). Continuous variables were summarized as median (interquartile range), and categorical variables as *n* (%). For Table 1, gender was summarized at the patient level: each patient was counted once within each study phase, and a patient contributing to more than one phase was counted once in each applicable phase. Age, BMI, weight, diagnosis, PCIS, laboratory measurements, and catheterization characteristics were analyzed at the catheterization level, retaining every catheterization. To account for repeated catheterizations within patients, continuous characteristics were compared using a cluster-adjusted rank-based Wald test and categorical characteristics using a Rao–Scott adjusted chi-square test, with the patient identifier as the clustering variable.

**Table 1:** Patient- and catheterization-level characteristics by study phase.

Characteristic	Baseline	Implementation	Follow-up	*p* value
A. Patient-level characteristics				
Patients, *n*	**121**	**76**	**63**	
Gender, *n* (%)				0.409
Female	56 (46.3)	31 (40.8)	23 (36.5)	
Male	65 (53.7)	45 (59.2)	40 (63.5)	
B. Catheterization-level characteristics				
Catheterizations, *n*	**170**	**105**	**100**	
Age, years	7.4 (2.8–11.6)	7.4 (3.8–11.9)	5.8 (2.0–10.9)	0.604
BMI, kg/m²	15.5 (13.9–18.5)	15.9 (13.8–17.9)	16.3 (14.2–17.8)	0.810
Weight, kg	23.5 (13.0–33.8)	18.0 (12.5–33.5)	18.0 (10.8–30.2)	0.518
Diagnosis, *n* (%)				0.433
Circulatory system diseases	32 (18.8)	13 (12.4)	13 (13.0)	
Neurological diseases	42 (24.7)	24 (22.9)	22 (22.0)	
Endocrine and metabolic diseases	5 (2.9)	2 (1.9)	8 (8.0)	
Hematologic and oncologic diseases	22 (12.9)	14 (13.3)	6 (6.0)	
Infectious diseases	5 (2.9)	8 (7.6)	4 (4.0)	
Respiratory system diseases	27 (15.9)	19 (18.1)	19 (19.0)	
Digestive system diseases	6 (3.5)	7 (6.7)	8 (8.0)	
Others	25 (14.7)	10 (9.5)	8 (8.0)	
Renal and immune system diseases	6 (3.5)	8 (7.6)	12 (12.0)	
PCIS	88 (82–90)	86 (78–90)	86 (80–88)	0.382
PLT, × 10^9^/L	219 (119–329)	198 (109–294)	230 (110–326)	0.598
INR	1.18 (1.09–1.38)	1.20 (1.11–1.42)	1.21 (1.10–1.33)	0.498
APTT, s	39.7 (34.7–49.2)	42.4 (34.6–49.4)	37.7 (34.1–41.7)	0.019*
MAP, mmHg	70.0 (60.0–80.6)	66.3 (58.7–76.0)	73.7 (63.5–88.7)	0.026*
Catheter insertion site, *n* (%)				0.652
Radial artery	137 (80.6)	75 (71.4)	77 (77.0)	
Dorsalis pedis artery	19 (11.2)	17 (16.2)	14 (14.0)	
Ulnar artery	8 (4.7)	10 (9.5)	8 (8.0)	
Other	6 (3.5)	3 (2.9)	1 (1.0)	
Catheter size, *n* (%)				0.482
22G	95 (55.9)	51 (48.6)	58 (58.0)	
24G	75 (44.1)	54 (51.4)	42 (42.0)	
Puncture method, *n* (%)				0.480
Blind puncture	134 (78.8)	75 (71.4)	76 (76.0)	
Ultrasound-guided	36 (21.2)	30 (28.6)	24 (24.0)	

APTT, activated partial thromboplastin time; BMI, body mass index; INR, international normalized ratio; MAP, mean arterial pressure; PCIS, pediatric critical illness score; PLT, platelet count.

Values are median (interquartile range) or *n* (%). Gender was summarized at the patient level: each patient was counted once within each study phase, and patients contributing data to more than one phase were counted once in each applicable phase. Age, BMI, weight, diagnosis, PCIS, laboratory measurements, and catheterization characteristics were summarized at the catheterization level, retaining every catheterization record. Overall *p* values account for within-patient clustering: continuous variables were compared using a cluster-adjusted rank-based Wald test, and categorical variables using a Rao–Scott adjusted chi-square test. Percentages use phase-specific denominators; missing values, if present, are shown separately.

Bold values indicate the phase-specific denominators (numbers of patients or catheterizations); *p < 0.05.

Statistical process control (SPC) charts were used as the primary longitudinal analysis: *P*-charts for adherence and first-attempt success and a *U*-chart for complication rates. Monthly denominators were the corresponding numbers of eligible audit opportunities or catheterizations, and variable control limits were calculated from the monthly subgroup sizes. Centerlines were calculated separately for the baseline and pooled post-intervention periods (implementation and follow-up) according to the prespecified study phases and were interpreted descriptively. Special-cause variation was assessed using Western Electric rules. Kendall's Tau assessed temporal trends. Secondary pairwise phase comparisons reported relative risks, risk differences, and 95% confidence intervals using the DescTools and epitools packages. A two-sided *p* value < 0.05 was considered statistically significant.

### Ethical considerations

The secondary use of data from this quality-improvement initiative for scholarly analysis and publication was subsequently reviewed and approved by the Ethics Committee of Shenzhen Children's Hospital (approval no. 202602501). The initiative was conducted as part of routine institutional quality-improvement activities using routinely collected clinical and audit data; accordingly, the committee waived the requirement for individual informed consent. All data were de-identified before analysis.

## Results

### Patient- and catheterization-level characteristics

Table 1 summarizes characteristics at two analytic levels. At the patient level, gender data included 121, 76, and 63 patients in the baseline, implementation, and follow-up phases, respectively. At the catheterization level, 170, 105, and 100 procedures were analyzed, respectively (375 total). No statistically significant phase differences were observed in gender, age, BMI, weight, diagnosis, PCIS, PLT, INR, catheter insertion site, catheter size, or puncture method (all *p* > 0.05). APTT differed across phases—39.7 (34.7–49.2), 42.4 (34.6–49.4), and 37.7 (34.1–41.7) s, respectively (*p* = 0.019)—as did MAP: 70.0 (60.0–80.6), 66.3 (58.7–76.0), and 73.7 (63.5–88.7) mmHg, respectively (*p* = 0.026).

### Overall nursing adherence

**Figure 2:**
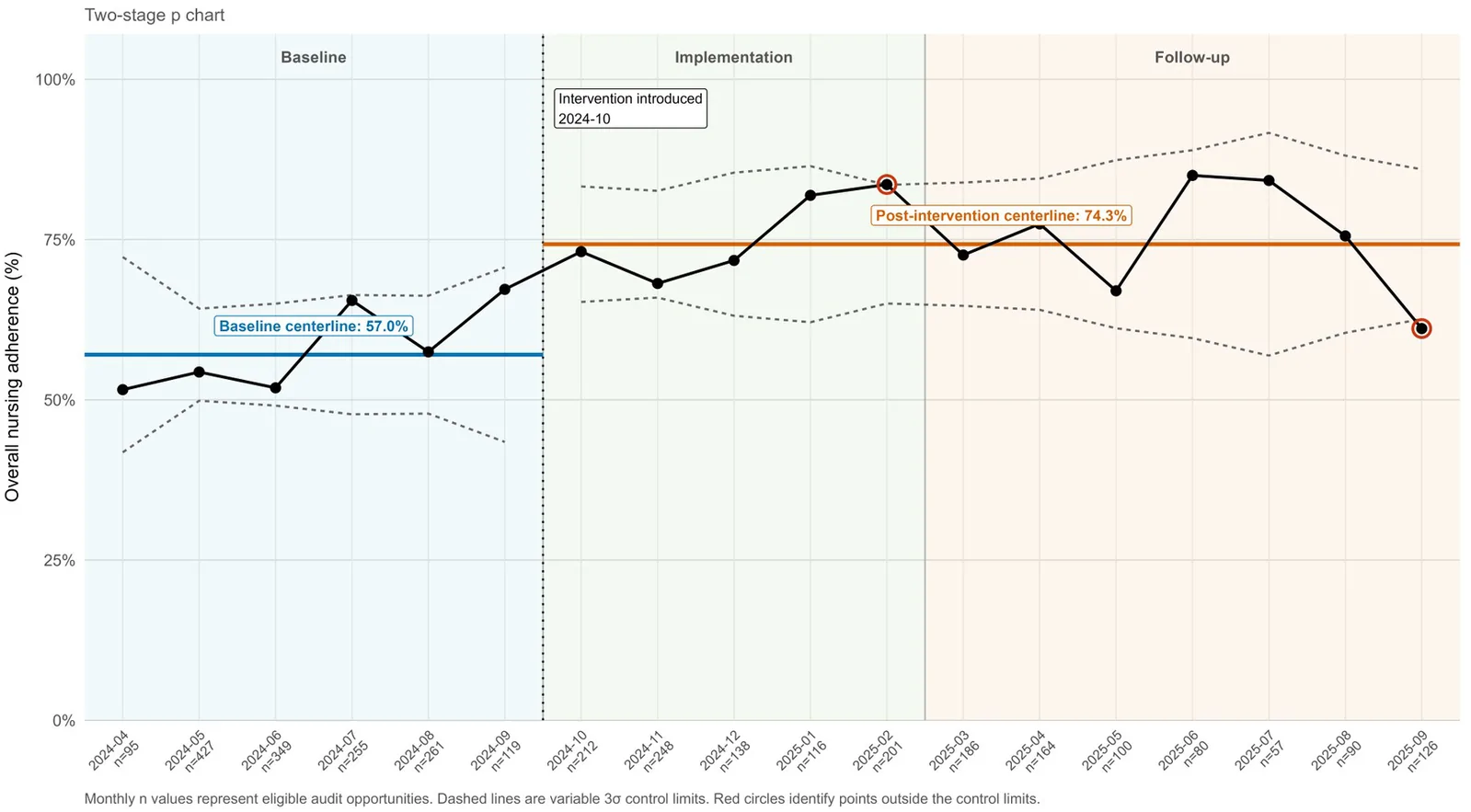
Overall nursing adherence.

Overall adherence improved after implementation, as shown in Figure 2. During baseline, adherence remained below 60%, with a centerline of 57.0% and wide control limits. After implementation, adherence exceeded 70% in most subsequent months; the shared implementation and follow-up centerline was 74.3%, with narrower control limits. Pairwise analysis showed a significant improvement from baseline to implementation (RR > 1, *p* < 0.001), with no statistically significant difference between implementation and follow-up (*p* > 0.05) (Supplementary Appendix Tables 3 and 4).

### Assessment adherence

**Figure 3:**
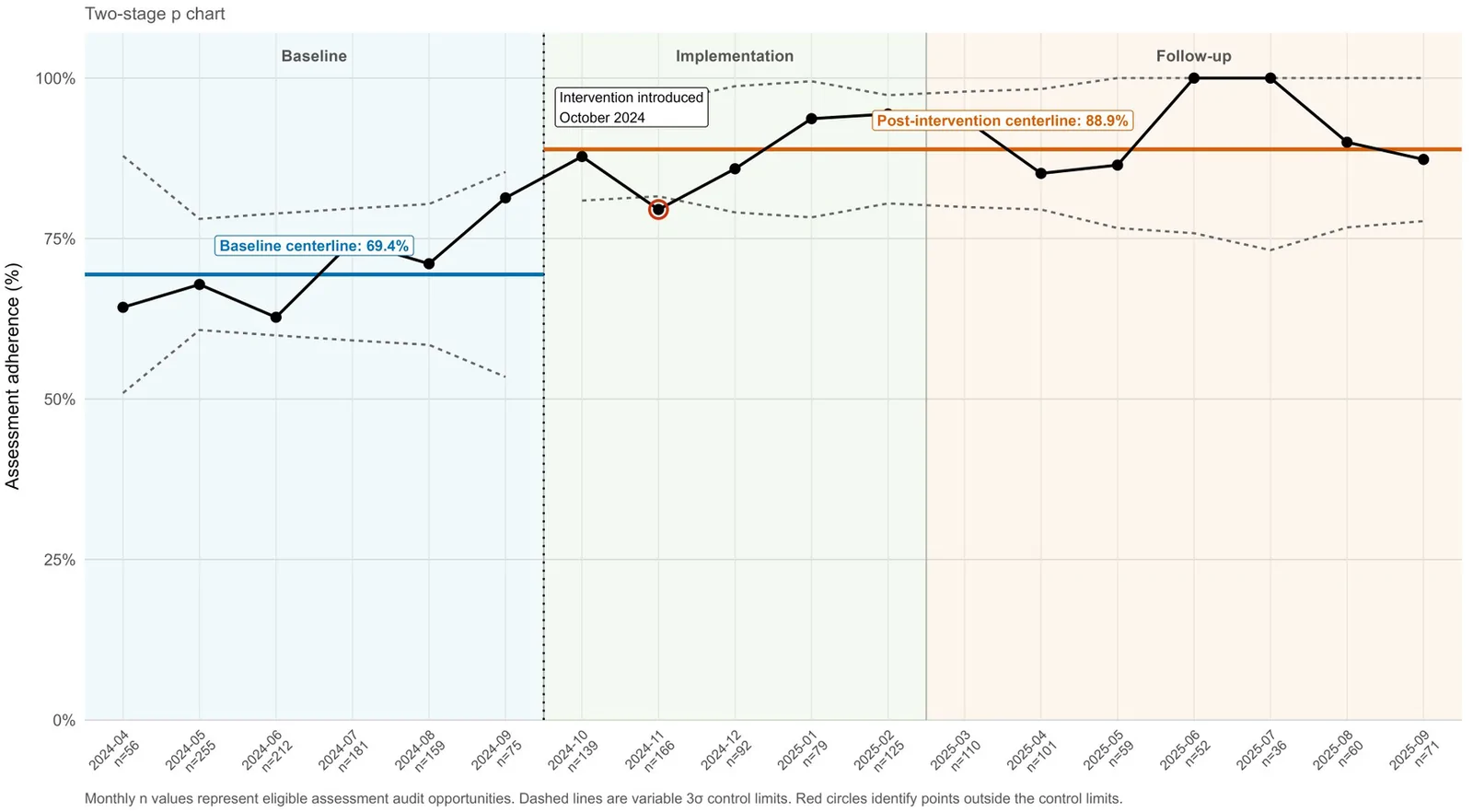
Assessment adherence.

Assessment adherence increased after implementation, as shown in Figure 3. During baseline, adherence fluctuated around a centerline of 69.4%. Post-implementation, adherence exceeded 85%, with a centerline of 88.9% and narrower control limits. The difference between the implementation and follow-up phases was not statistically significant (*p* = 0.072) (Supplementary Appendix Tables 3 and 4).

### Insertion adherence

**Figure 4:**
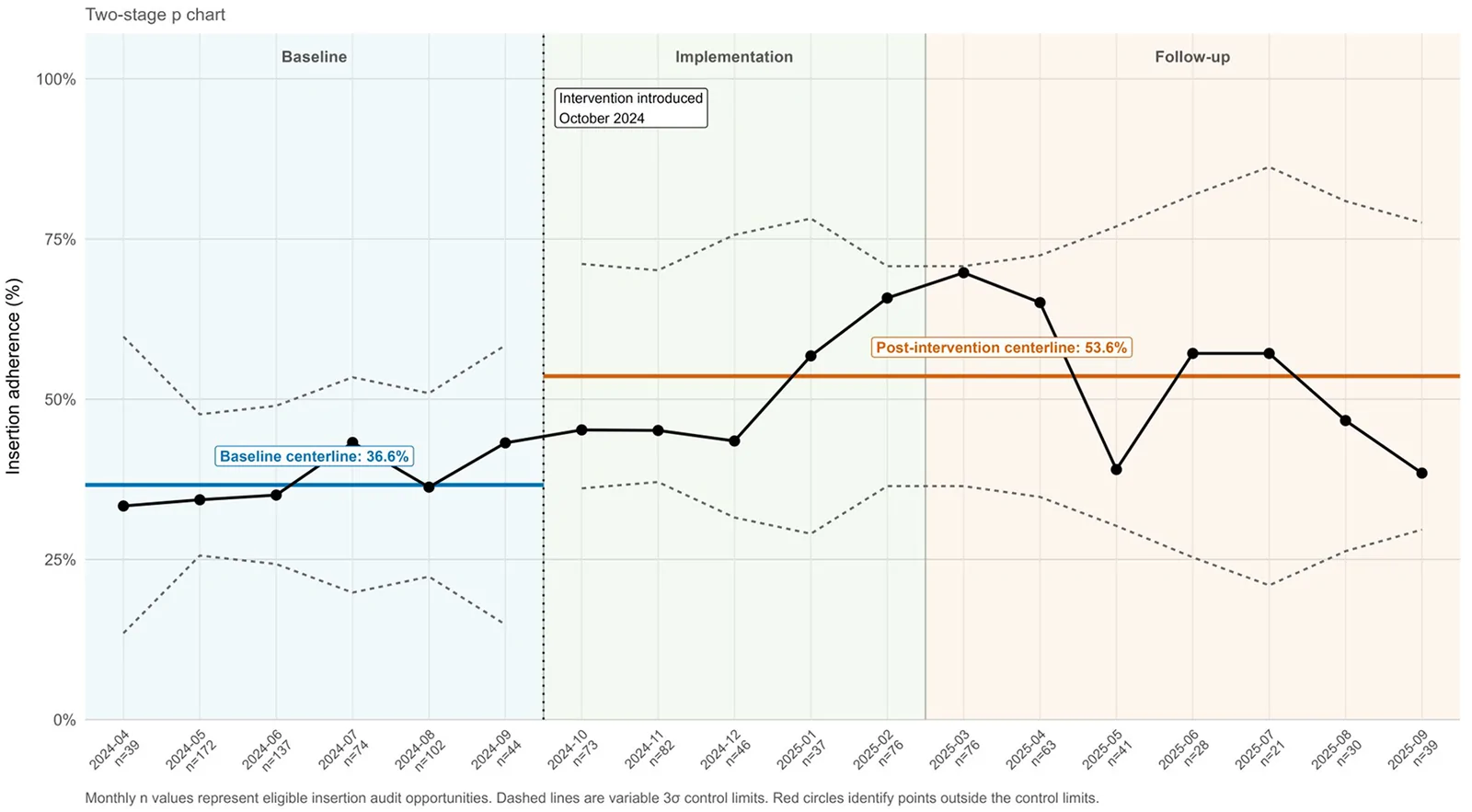
Insertion adherence.

Insertion adherence improved from a baseline centerline of 36.6% to a post-implementation centerline of 53.6%, as shown in Figure 4. Control limits remained wider, with occasional downward fluctuations. Pairwise comparisons showed significant improvement from baseline (*p* < 0.01), with no statistically significant difference between implementation and follow-up (*p* = 0.237) (Supplementary Appendix Tables 3 and 4).

### First-attempt success rate

**Figure 5:**
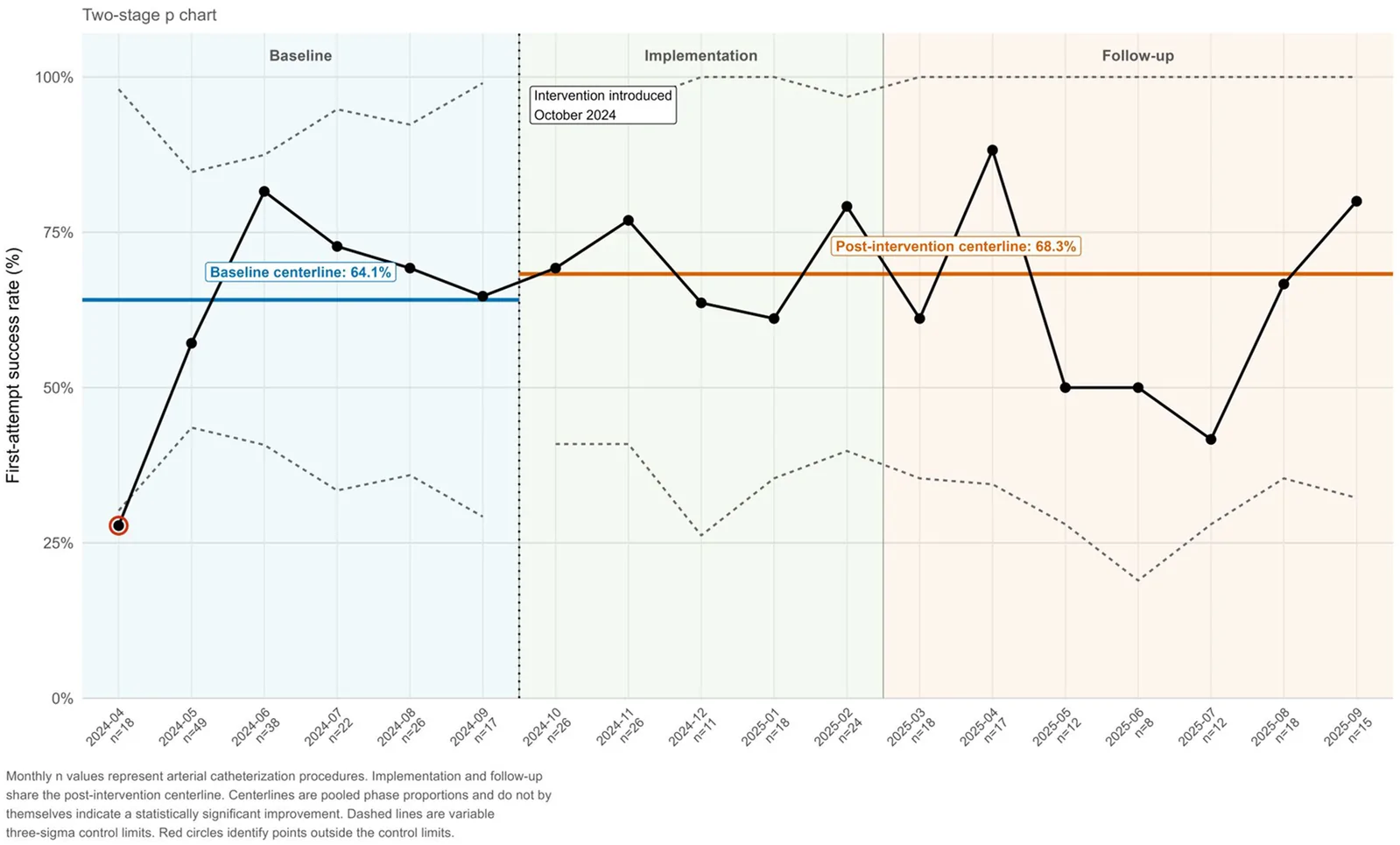
First-attempt success rate.

The first-attempt success rate remained variable across the study period, as shown in Figure 5. The baseline centerline was 64.1%, compared with 68.3% post-implementation—an absolute increase of 4.2 percentage points. However, the chart did not meet the prespecified SPC criteria for a sustained process shift, and no pairwise phase comparison was statistically significant (all *p* > 0.20) (Supplementary Appendix Tables 3 and 4).

### Complication rate

**Figure 6:**
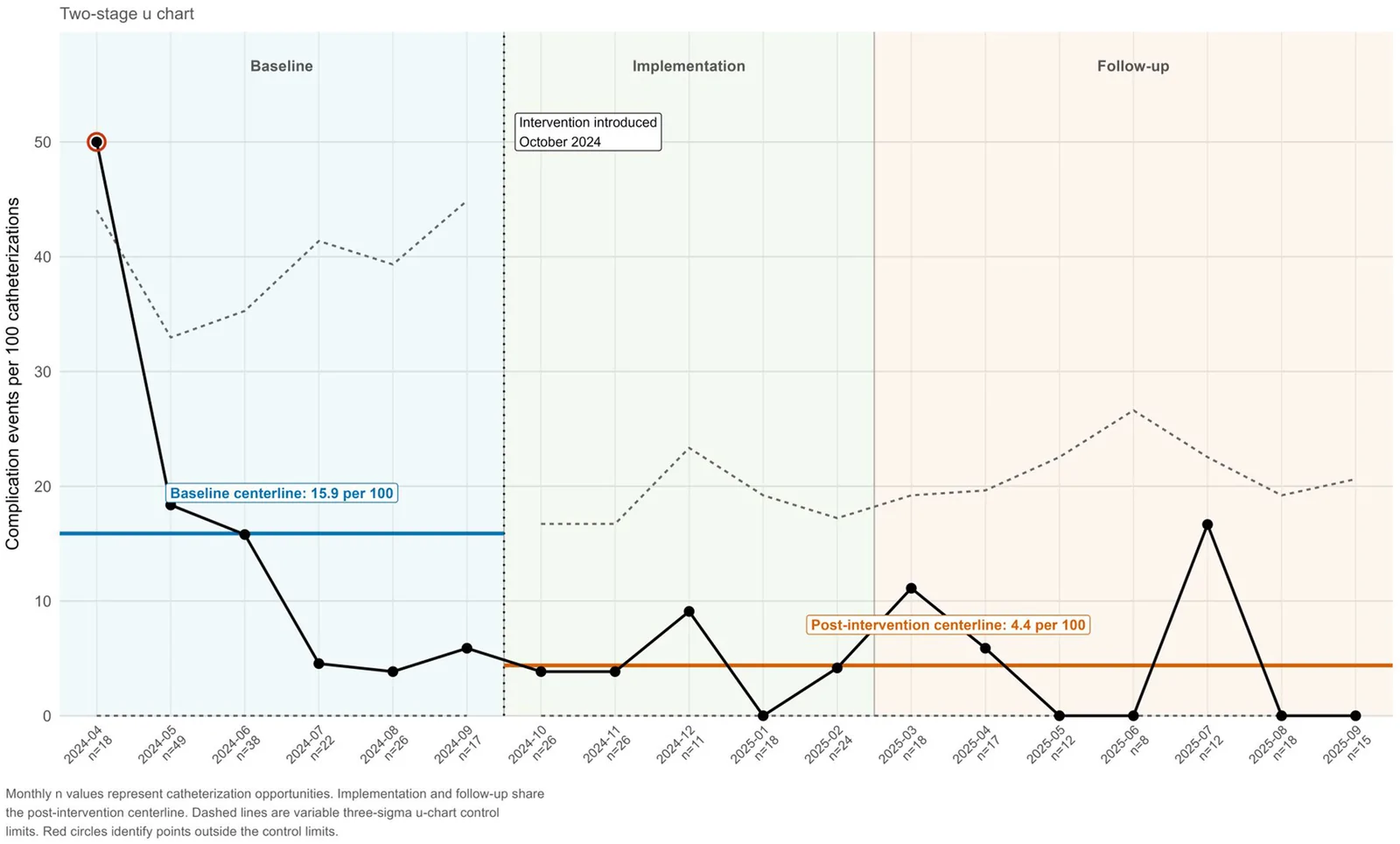
Arterial catheter complication event rate.

Complication rates declined in temporal association with implementation, as shown in Figure 6. During baseline, monthly rates were variable, with some months exceeding 40%; post-implementation rates were generally lower. Trend analysis showed a significant downward trend (Kendall's Tau = −0.432, *p* = 0.017). Unadjusted pairwise comparisons indicated lower complication rates in the post-implementation phases (RR < 0.50, *p* < 0.01), with no statistically significant difference between implementation and follow-up (*p* = 0.743) (Supplementary Appendix Table 5).

## Discussion

This study observed that structured process interventions—checklists, pre-procedural verification, and audit feedback—were associated with higher procedural adherence and fewer observed complications in pediatric PAC management (18, 19). Assessment adherence increased rapidly and remained stable through follow-up, consistent with studies showing that standardized workflows and bedside prompts reduce process variability (18, 20). The concurrent decline in complications is consistent with the proposition that process reliability may support safer vascular access (21), but temporal concurrence alone does not establish a causal effect. Standardizing PAC assessment and insertion nevertheless remains clinically important because bleeding, hematoma, and other localized complications are potentially preventable (22).

The gap between assessment and insertion outcomes deserves attention. Assessment adherence rose quickly because it involves cognitive and procedural steps—checklists, verification, documentation—that respond well to standardization. Insertion adherence improved only moderately and remained variable. This is consistent with evidence that, without targeted competency-based interventions, PAC insertion remains operator-dependent and prone to failure (22, 23). Procedural failure can occur at multiple points: inadequate evaluation of coagulation status or collateral circulation before insertion increases complication risk (7); inaccurate vessel localization and incorrect needle angle are major causes of unsuccessful attempts during insertion (4, 22); suboptimal fixation and non-standardized post-procedural care add further risk (3, 14).

Randomized trials have shown that ultrasound guidance—particularly the long-axis in-plane approach or dynamic needle-tip positioning—can improve first-attempt and overall success and shorten insertion time (24). Recent multidisciplinary guidance supports ultrasound use to standardize peripheral arterial puncture and reduce operator variability (25). In the present study, however, first-attempt success increased by only 4.2 percentage points, with no sustained SPC shift or statistically significant phase difference. This null result may reflect the learning curve for ultrasound-guided techniques, heterogeneity in operator experience and case complexity, or insufficient intervention exposure. Because operator-level performance and actual ultrasound use were not measured, the relative contribution of these factors cannot be determined.

The pattern observed here—rapid improvement followed by a plateau—is common in quality improvement work (26). Once a system reaches a high-reliability benchmark, further gains require different strategies: competency development, technical refinement, or both (27). Simply increasing audit frequency or reinforcing checklists has limited marginal benefit when adherence approaches saturation. For PAC insertion, the next steps should include competency-based simulation training and routine ultrasound guidance, which have been shown to increase success rates, shorten insertion time, and reduce complications (28, 29).

The intervention itself—checklists, audits, feedback, and tiered training—is straightforward and low-cost. These components are adjustable to different institutional capabilities and resource levels, making the approach scalable to other PICUs. Future research should examine whether ultrasound use and procedural fidelity mediate first-attempt success and complication rates, which would help refine intervention design.

### Limitations

This study has limitations inherent to a single-site, uncontrolled pre-post design. Without a concurrent control group, secular trends, staffing or workflow changes, case-mix variation, maturation, and observation effects may have contributed to the findings. Although SPC strengthened longitudinal assessment and Table 1 comparisons accounted for repeated catheterizations within patients, neither approach eliminates confounding. APTT and MAP differed significantly across phases and are mechanistically relevant to bleeding, hematoma, hemodynamic status, and vascular perfusion. Because the complication comparisons were not adjusted for these covariates, the observed reduction cannot be attributed solely to the intervention.

Phase-specific patient counts were not mutually exclusive because some patients underwent catheterization in more than one phase, and repeated catheterizations were retained in catheterization-level analyses. The modest sample size and single-center setting limit generalizability to PICUs with different case-mix, workforce competency, organizational culture, or resources. Intervention fidelity, operator skill acquisition, and ultrasound proficiency were not quantified, leaving the mechanisms underlying the observed process changes uncertain. Future studies should use controlled, multi-center or interrupted time-series designs, incorporate patient-clustered adjusted outcome models, and measure implementation fidelity and operator competency directly.

## Conclusion

This single-center quality improvement study suggests that standardizing PAC assessment and insertion processes is associated with improved nursing adherence and lower observed complication rates. The absence of a concurrent control group and phase differences in APTT and MAP prevent definitive causal attribution for the complication outcome. Assessment processes responded more readily than operator-dependent insertion performance, and first-attempt success did not demonstrate a sustained improvement. Future controlled studies should evaluate routine ultrasound use, competency-based training, procedural fidelity, and patient-clustered adjusted safety outcomes.

## Data Availability

The datasets supporting the findings of this study are not publicly available because they contain clinical data subject to institutional and ethical restrictions. De-identified data may be made available by the corresponding author upon reasonable request and with institutional approval. Requests should be directed to Linjuan Wang (wanglinjuan0111@126.com).
