# Supplementary material for: Improving peripheral arterial catheter placement in critically ill children: a pre-post quality improvement study
Source: Front Pediatr. 2026 Aug 25;14:1931589. doi: 10.3389/fped.2026.1931589 (PMC13550120; doi:10.3389/fped.2026.1931589)
Supplement: Supplementary file 1 [file Supplementaryfile1.docx]

**Appendix Table 1 Evidence of placement of PACs in PICU and clinical review indicators**

| **Evidence** | **Clinical audit criteria** | **Audit methods** |
| --- | --- | --- |
| 1. Indications for indwelling arterial catheterization: Continuous monitoring of arterial blood pressure is required; Continuous monitoring of hemodynamics is required to predict volume response and guide rescue and treatment; Frequently collect blood samples. Contraindications: Local infection, thrombosis, or anatomical changes at the puncture site; Severe peripheral vascular disease or active Raynaud's syndrome, etc. (Grade B) | C1: Indwelling arterial line meets the indications and contraindications of arterial line | On-site inspection or documentation or knowledge questionnaire |
| 2 The platelet count, international normalized ratio, and activated partial thromboplastin time of the child should be checked before indwelling the arterial catheter. Arterial catheterization can be performed when platelet count ≥50×10^9^/L; Repeated arterial puncture should be avoided when the international normalized ratio ≥3 and/or activated partial thromboplastin time ≥100 s (Grade B) | C2: Check the child's coagulation indicators and platelet count before performing arterial puncture | On-site inspection |
|  | C3: Children with PLT≥50×10^9^/L who underwent arteriocatheter indwelling | On-site inspection or documentation |
|  | C4: INR≥3 or /APTT≥100s in the same vessel puncture ≤3 times | On-site inspection |
| 3. Before radial artery catheterization, the Allen test, modified Allen test, or Doppler flow detector should be used to assess collateral circulation of the artery (Grade B). | C5: Perform the Allen test or modified Allen test to check collateral circulation before radial artery puncture | On-site inspection |
| 4. For critically ill children, radial artery, posterior tibial artery and dorsal foot artery catheterization can be used, but brachial artery catheterization cannot be used due to the lack of collateral circulation. Among them, the radial artery is the preferred site for catheter placement (Grade B) | C6: Nurse selection of arterial catheterization site, radial artery > posterior tibial artery, dorsal foot artery, brachial artery catheterization is not allowed | On-site inspection or document record |
| 5 Select the appropriate type of arterial catheter based on the child's weight. For children weighing less than 10kg, 24 G or 22 G arterial catheters can be used; For children weighing 10 to 40 kg, 22 G arterial catheters can be used; For children weighing more than 40 kg, 22 G or 20 G arterial catheters may be used (Grade B) | C7: Arterial catheter type selection: <10 kg: 24 G or 22 G; 10-40 kg: 22 G; >40 kg: 22 G or 20 G | On-site inspection or documentation |
| 6. Arterial catheters can only be indwelled by trained medical staff (Grade B) | C8: An arterial catheter should be indwelled by a healthcare professional who is qualified for arterial puncture | On-site inspection or documentation |
| 7. Recommended catheterization under ultrasound guidance (Grade A) | C9: Ultrasound-guided arterial indwelling | On-site inspection |
| 8. When inserting a peripheral arteriocatheter, a cap, mask, sterile gloves and goggles should be worn, and sterile hole wipes should be used (Grade B) | C10: Wear a cap, mask, sterile gloves and goggles (if necessary) during arterial indwelling, and use a sterile orifice towel | On-site inspection |
| 9. Topical skin disinfection using povidone-iodine, 70% ethanol or 2% chlorhexidine gluconate solution (Grade A) | C11: During arterial puncture, local skin disinfection is carried out using povidone-iodine, 70% ethanol or 2% chlorhexidine gluconate solution | On-site inspection |
| 10. During the arterial puncture catheterization of a conscious child, local surface anesthetics (such as lidocaine liposome ointment or lidocaine-propacaine cream), sedative drugs, or appropriate limb restraint can be used to control the child's pain and limit movement to improve the safety of the catheterization, but it should be noted that excessive restraint may lead to weakened arterial pulsation in the child. Thereby reducing the success rate of puncture (Grade B) | C12: Nurses perform arterial indwelling on conscious children with local surface anesthetic/sedation/restraint | On-site inspection |

**Appendix Table 2: GRiP Matrix: Barriers, Strategies, and Resources for Implementing Evidence-Based PAC Practices in Pediatric Intensive Care**

| **Barrier Type** | **Identified Barrier** | **Audit Criterion** | **Strategy to Address Barrier** | **Resources Required** |
| --- | --- | --- | --- | --- |
| Context – Structural | Lack of portable ultrasound machines | C9 | • Procure portable devices | Budget, supply chain, technical support |
|  | Shortage of catheter-related supplies (e.g., drapes, 2% chlorhexidine) | C10, C11 | • Stock audit and procurement optimization  • Establish consumable inventory and alarm system | Logistics & inventory team |
|  | No integration of decision tools into EMR | C2, C3, C4 | • Develop and embed a coagulation assessment decision tree with alerts | EMR developer, nurse informatics |
| Context – Process | No mandatory collateral circulation or coagulation assessment | C5, C2, C7 | • Revise catheterization process flow  • Enforce assessment prior to insertion  • Add selection criteria for catheter types | Protocol committee, clinical educators |
|  | No regular evaluation of PAC-related outcomes | C2, C5, C6, C7, C9 | • Establish a data-driven feedback loop  • Form quality review taskforce | Audit team, data analysis tools |
| Innovation – Complexity | Complex logic for coagulation risk (INR/APTT/platelets) | C2, C3, C4 | • Develop simplified score-based decision aid  • Deliver targeted online training | Clinical algorithm, learning management system platform |
|  | Ultrasound or vascular assessments require specific training | C6 | • Create practice-based competency modules | Simulation center, nurse trainers |
| Innovation – Compatibility | Ultrasound guidance delays emergency response | C9 | • Pre-position devices and kits  • Use staged training to reduce operation time |  |
|  | Time pressure leads to skipped steps (e.g., Allen test, disinfection) | C5, C8, C11, C12 | • Introduce checklist-based monitoring  • Use simulation to reinforce completeness | Printed checklists, simulation drills |
| Adopter – Knowledge/Skill Gaps | Inadequate ultrasound-guided puncture skills | C9 | • Apply tiered training: simulation → assessment → supervision  • Assign experienced mentors and shared device use | Training program, senior PICU nurses |
|  | Lack of pediatric pain management techniques | C12 | • Provide training on surface anesthesia  • Emphasize comfort positioning and reduce restraint | Education sessions, pain scale tools |
| Adopter – Attitudes/Beliefs | Perception that ultrasound not needed for “easy cases” | C9 | • Use monthly complication data review  • Link nurse performance to patient outcomes | Quality dashboard, recognition program |
|  | Low perception of benefit from pre-assessment | C2, C5 | • Use real case discussions and visual storytelling to enhance awareness | Monthly meeting slots, visual case library |
|  | Resistance to new disinfection protocols | C10 | • Peer modeling and role reinforcement from senior nurses | Clinical leaders, feedback culture |

**Appendix Table 3：Pairwise results of group comparisons**

| **metric** | **contrast** | **RR** | **RR_l** | **RR_u** | **RD** | **RD_l** | **RD_u** | **p_value** |
| --- | --- | --- | --- | --- | --- | --- | --- | --- |
| Overall nursing adherence | Baseline vs Change | 0.583 | 0.513 | 0.661 | 0.179 | 0.142 | 0.217 | 0.000 |
| Overall nursing adherence | Baseline vs Follow-up | 0.617 | 0.543 | 0.702 | 0.164 | 0.125 | 0.204 | 0.000 |
| Overall nursing adherence | Change vs Follow-up | 1.060 | 0.903 | 1.245 | -0.015 | -0.057 | 0.027 | 0.479 |
| Assessment adherence | Baseline vs Change | 0.413 | 0.328 | 0.521 | 0.180 | 0.140 | 0.219 | 0.000 |
| Assessment adherence | Baseline vs Follow-up | 0.301 | 0.224 | 0.404 | 0.214 | 0.175 | 0.253 | 0.000 |
| Assessment adherence | Change vs Follow-up | 0.728 | 0.513 | 1.031 | 0.034 | -0.003 | 0.071 | 0.072 |
| insertion adherence | Baseline vs Change | 0.769 | 0.675 | 0.875 | 0.147 | 0.079 | 0.215 | 0.000 |
| insertion adherence | Baseline vs Follow-up | 0.694 | 0.601 | 0.800 | 0.194 | 0.125 | 0.263 | 0.000 |
| insertion adherence | Change vs Follow-up | 0.902 | 0.760 | 1.071 | 0.048 | -0.031 | 0.127 | 0.237 |
| First-attempt success rate | Baseline vs Change | 0.796 | 0.554 | 1.145 | 0.073 | -0.039 | 0.186 | 0.211 |
| First-attempt success rate | Baseline vs Follow-up | 0.975 | 0.698 | 1.363 | 0.009 | -0.109 | 0.127 | 0.884 |
| First-attempt success rate | Change vs Follow-up | 1.225 | 0.818 | 1.834 | -0.064 | -0.192 | 0.063 | 0.323 |
| Arterial catheter complication rate | Baseline vs Change | 1.144 | 1.060 | 1.233 | -0.121 | -0.187 | -0.055 | 0.002 |
| Arterial catheter complication rate | Baseline vs Follow-up | 1.129 | 1.043 | 1.223 | -0.109 | -0.178 | -0.039 | 0.008 |
| Arterial catheter complication rate | Change vs Follow-up | 0.988 | 0.931 | 1.048 | 0.012 | -0.044 | 0.068 | 0.743 |

**Table 4 Stage summary wilson of group comparisons**

| **phase** | **x** | **n** | **p_hat** | **lcl** | **ucl** | **percent** | **metric** |
| --- | --- | --- | --- | --- | --- | --- | --- |
| Baseline | 859 | 1506 | 0.570 | 0.545 | 0.595 | 57.0 | Overall nursing adherence |
| Change | 686 | 915 | 0.750 | 0.721 | 0.777 | 75.0 | Overall nursing adherence |
| Follow-up | 590 | 803 | 0.735 | 0.703 | 0.764 | 73.5 | Overall nursing adherence |
| Baseline | 651 | 938 | 0.694 | 0.664 | 0.723 | 69.4 | Assessment adherence |
| Change | 525 | 601 | 0.874 | 0.845 | 0.898 | 87.4 | Assessment adherence |
| Follow-up | 444 | 489 | 0.908 | 0.879 | 0.931 | 90.8 | Assessment adherence |
| Baseline | 208 | 568 | 0.366 | 0.328 | 0.407 | 36.6 | insertion adherence |
| Change | 161 | 314 | 0.513 | 0.458 | 0.568 | 51.3 | insertion adherence |
| Follow-up | 167 | 298 | 0.560 | 0.504 | 0.616 | 56.0 | insertion adherence |
| Baseline | 109 | 170 | 0.641 | 0.567 | 0.709 | 64.1 | First-attempt success rate |
| Change | 75 | 105 | 0.714 | 0.622 | 0.792 | 71.4 | First-attempt success rate |
| Follow-up | 65 | 100 | 0.650 | 0.553 | 0.736 | 65.0 | First-attempt success rate |
| Baseline | 27 | 170 | 0.159 | 0.112 | 0.221 | 15.9 | Arterial catheter complication rate |
| Change | 4 | 105 | 0.038 | 0.015 | 0.094 | 3.8 | Arterial catheter complication rate |
| Follow-up | 5 | 100 | 0.050 | 0.022 | 0.112 | 5.0 | Arterial catheter complication rate |

**Table 5 Trend test of groups**

| **stat** | **p_value** | **metric** | **tau** |
| --- | --- | --- | --- |
| NA | NA | Overall nursing adherence | NA |
| NA | NA | Assessment adherence | NA |
| NA | NA | insertion adherence | NA |
| NA | NA | First-attempt success rate | NA |
| NA | 0.017 | Arterial catheter complication rate per 100 cases | -0.432 |
| NA: trend cannot be calculated | | | |
